# The Effect of Sensor Placement and Number on Physical Activity Recognition and Energy Expenditure Estimation in Older Adults: Validation Study

**DOI:** 10.2196/23681

**Published:** 2021-05-03

**Authors:** Anis Davoudi, Mamoun T Mardini, David Nelson, Fahd Albinali, Sanjay Ranka, Parisa Rashidi, Todd M Manini

**Affiliations:** 1 Department of Biomedical Engineering University of Florida Gainesville, FL United States; 2 Department of Aging and Geriatric Research University of Florida Gainesville, FL United States; 3 Qmedic Medical Alert Systems Boston, MA United States; 4 Department of Computer and Information Science and Engineering University of Florida Gainesville, FL United States

**Keywords:** human activity recognition, machine learning, wearable accelerometers, mobile phone

## Abstract

**Background:**

Research has shown the feasibility of human activity recognition using wearable accelerometer devices. Different studies have used varying numbers and placements for data collection using sensors.

**Objective:**

This study aims to compare accuracy performance between multiple and variable placements of accelerometer devices in categorizing the type of physical activity and corresponding energy expenditure in older adults.

**Methods:**

In total, 93 participants (mean age 72.2 years, SD 7.1) completed a total of 32 activities of daily life in a laboratory setting. Activities were classified as sedentary versus nonsedentary, locomotion versus nonlocomotion, and lifestyle versus nonlifestyle activities (eg, leisure walk vs computer work). A portable metabolic unit was worn during each activity to measure metabolic equivalents (METs). Accelerometers were placed on 5 different body positions: wrist, hip, ankle, upper arm, and thigh. Accelerometer data from each body position and combinations of positions were used to develop random forest models to assess activity category recognition accuracy and MET estimation.

**Results:**

Model performance for both MET estimation and activity category recognition were strengthened with the use of additional accelerometer devices. However, a single accelerometer on the ankle, upper arm, hip, thigh, or wrist had only a 0.03-0.09 MET increase in prediction error compared with wearing all 5 devices. Balanced accuracy showed similar trends with slight decreases in balanced accuracy for the detection of locomotion (balanced accuracy decrease range 0-0.01), sedentary (balanced accuracy decrease range 0.05-0.13), and lifestyle activities (balanced accuracy decrease range 0.04-0.08) compared with all 5 placements. The accuracy of recognizing activity categories increased with additional placements (accuracy decrease range 0.15-0.29). Notably, the hip was the best single body position for MET estimation and activity category recognition.

**Conclusions:**

Additional accelerometer devices slightly enhance activity recognition accuracy and MET estimation in older adults. However, given the extra burden of wearing additional devices, single accelerometers with appropriate placement appear to be sufficient for estimating energy expenditure and activity category recognition in older adults.

## Introduction

### Background

Over the past 30 years, accelerometer devices have been widely used for measuring movements, physical activity categories, and energy expenditure [1]. This work has also carried forward into characterizing the activity patterns of patients with chronic diseases such as obesity, cardiovascular disease, schizophrenia, bipolar disorder, and cancer [2-6]. Despite its growing use in both clinical and research settings, the optimal body position for sensor placement that would provide the most accurate activity category recognition and the corresponding estimate of energy expenditure in older adults remains uncertain. For example, previous studies have used various sensor placements on the body, including the wrist [7-9], thigh [10,11], hip [12-14], arm [15,16] or ankle [17,18], or a combination of multiple placements [19,20]. However, such studies have often been conducted on relatively small samples of young and middle-aged adults. There continues to be a gap in knowledge regarding body placement for older adults (>60 years). Such knowledge is important for considering older age as a factor for estimating activity types and energy expenditure.

There is a lack of a comprehensive evaluation that directly compares individual and combinations of accelerometers placed on different body positions. Historically, the hip position was chosen in both research and public settings for tracking steps (ie, steps per day). The hip position is close to the body’s center of the mass and provides an acceleration change because of the foot fall action-reaction when ambulating. As such, the hip position offers a convenient and accurate approach for capturing ambulatory activity [21]. The ankle position is also accurate in assessing step counts and other gait-related features [22-25]. Recently, however, the wrist position has become popular for collecting accelerometer data because of the increased prevalence of smartwatches. This is due to their convenience, ability to capture sleep quality, determination of 24-hour activity rhythms, and enhanced compliance [26-30].

### Objectives

A systemic evaluation of body placements will help optimize energy expenditure estimation and activity recognition. It would also help resolve controversies related to the balance between the accuracy and convenience of different body placements [31]. Given the paucity of information about the role of accelerometer placement on older adults, we aimed to compare and contrast energy expenditure estimation, individual activity, and activity category recognition with 5 sensor body positions and their combinations during 32 activities that included sedentary, locomotion, and lifestyle categories. We hypothesized that combined data from 5 accelerometer positions on the body would provide optimal energy expenditure estimation, individual activity recognition, and activity category recognition, but this improvement will be incremental compared with a single or combination of body placements.

## Methods

### Study Design

This study was approved by the University of Florida Institutional Review Board, and written informed consent was obtained from all participants. The inclusion criteria were designed to optimize safety while ensuring population representation. It included older adults, aged ≥60 years [32], with stratified enrollment for both high and low function according to scores on the standardized Short Physical Performance Battery [33]. The study pre-planned to enroll and complete testing in 90 participants with 30% (27/90) of the participants scoring in the lowest quartile of physical function. Recruitment focused on enrolling community-dwelling adults without significant health issues that could impact the safety of participants. Additional inclusion criteria included willingness to undergo all testing procedures, stable weight for at least 3 months, and ability to understand and speak English. Participants were excluded if they met any of the following criteria: failure to provide informed consent, use of a walker, lower extremity amputation, history of chest pain or severe shortness of breath during physical stress, poststroke syndrome causing ambulatory deficits, and requiring assistance with basic activities of daily living or living in a complete care nursing home. A complete list of the exclusion criteria can be found elsewhere [34].

### Accelerometers and Energy Expenditure During Activities

**Figure 1:**
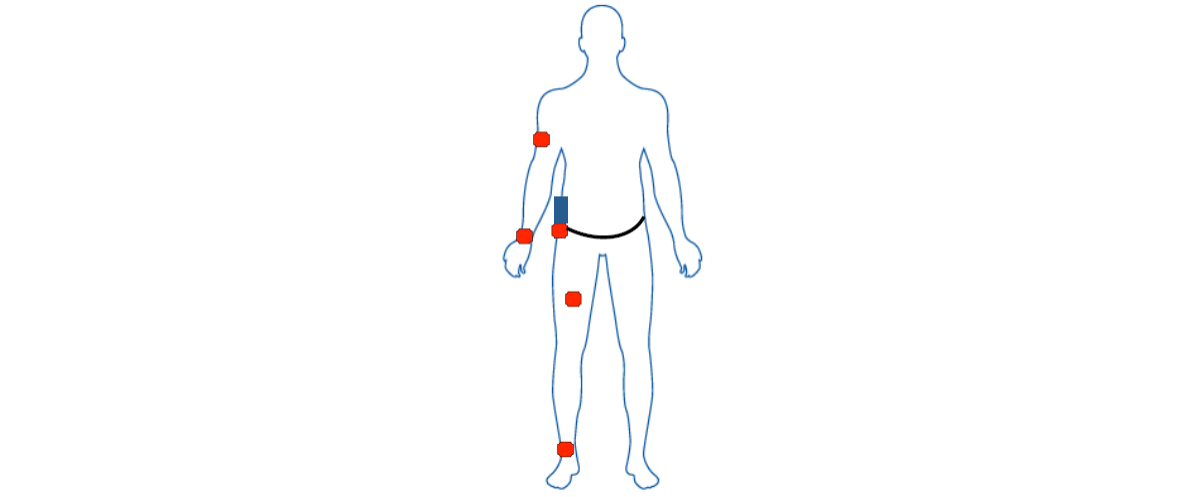
Sensor placement on the body.

Participants were asked to perform 32 scripted activities listed in Multimedia Appendix 1. These activities were chosen because they are common among most Americans and are consistent with the average time spent in the 2010 American Time Use Survey [35]. Activities were performed for 6 to 8 minutes with 5 to 10 minutes of rest between each activity. Assessments were completed over 4 separate visits. The participants received instructions from the research staff before each activity. Participants wore 5 ActiGraph GT3X triaxial accelerometers [36], one on their ankle, upper arm, hip, thigh, and wrist. All monitors were worn on the right side for the duration of data collection, as shown in Figure 1. Of note, Buchan et al [37] and Dieu et al [38] demonstrated strong agreement between accelerometer data collected on the dominant and nondominant sides. Accelerometers were initialized simultaneously and programmed to collect data at 100 Hz.

Participants wore a COSMED K4b2 [39] portable gas analysis system while performing the 32 scripted activities. Before data collection, the oxygen (O_2_) and carbon dioxide (CO_2_) sensors were calibrated using a gas mixture sample of 16.0% O_2_ and 5.0% CO_2_ and room air calibration. The turbine flow meter was calibrated using a 3.0-L syringe. A flexible facemask was positioned over the participant’s mouth and nose and attached to the flow meter. Oxygen consumption (VO_2_; measured in mL min^-1^ kg^-1^) was measured breath-by-breath, and data were subsequently smoothed with a 30-second running average window. VO_2_ data were displayed and manually evaluated to determine when steady-state VO_2_ was reached. A steady state was defined as a plateau in VO_2_, which typically occurs 2 minutes after the start of the activity. Data were expressed as metabolic equivalents (METs) after dividing the VO_2_ values by the traditional standard of 3.5 mL min^-1^ kg^-1^ [40]. A dedicated study smartphone with a custom-built app was synchronized to server time and used to record the start and stop times for each activity (shown in blue in Figure 1). This ensured that time windows could be accurately identified from accelerometer data that was also initialized to server time.

### Analysis

**Figure 2:**
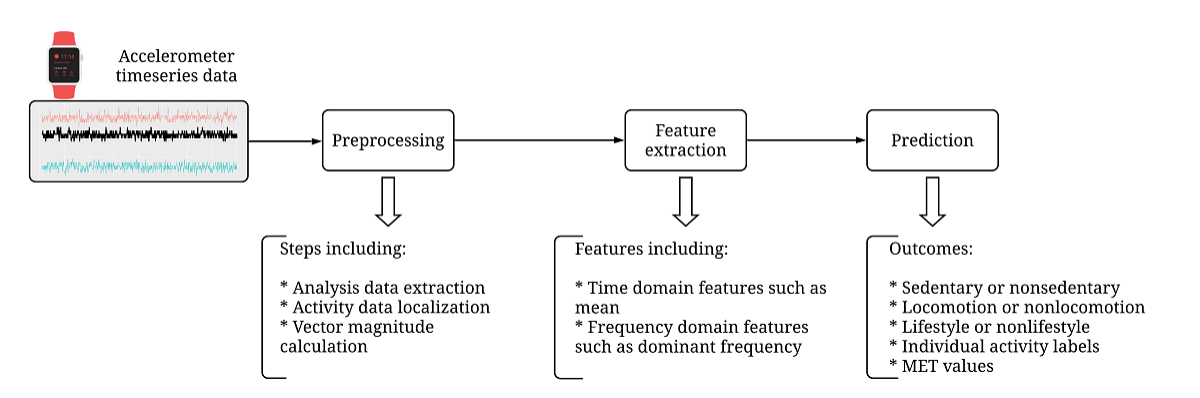
Analysis flow steps. After accelerometer data were downloaded using the ActiLife (ActiGraph) toolbox, preprocessing steps and feature extraction steps were completed to prepare the data set to be used in prediction models for each task. MET: metabolic equivalent.

Data were first processed to extract relevant summary features from each contiguous 16-second window. The features described in Table 1 represent both the time and frequency domains [41,42]. These features were included in the analytic models, as illustrated in the analysis flow in Figure 2. There were a total of 31 different wrist, hip, ankle, upper arm, and thigh body position combinations. The analyses compared the performance of single placement and combinations of device placements for estimating METs and for labeling activities as individual and when they were categorized as sedentary, locomotion, or lifestyle (Multimedia Appendix 1). We used random forest as our primary analysis approach, which is a frequently used machine learning algorithm, to recognize human activity from accelerometer data [41-45]. Random forest is an ensemble learning algorithm that builds a large number of decision trees from random sub–data sets of the training data set. The predicted class is determined by aggregating the predicted classes (votes) from the individual decision trees and selecting the majority class in case of classification or by averaging the predicted values in case of regression [46]. This procedure was first performed to evaluate the accuracy of detecting activity categories based on sedentary versus nonsedentary, locomotion versus nonlocomotion, and lifestyle versus nonlifestyle activities as well as to evaluate the accuracy of classifying each of the 32 individual activities against a 3.1% random chance of matching correctly. We used a regression random forest for continuous MET estimation and classification of random forest for activity recognition. To reduce bias, the data were split randomly into development and testing data sets using participant identification numbers. Participants were included in either the development or testing data sets but not both. The development data set was further randomly split into training and validating data sets to tune the model parameters. Nested cross-validation was used; in each outer fold, we kept five-sixths of the participants for model development and one-sixth of the participants for testing. In each inner fold, four-fifths of the participants in the development data set were assigned to the training data set, and one-fifth of the participants were assigned to the validating data set. All model estimates were reported for the testing data sets. In supplementary analyses, a confusion matrix of actual versus predicted activities (32×32 matrix) from the hip and wrist positions, respectively, was generated to help interpret the accuracy and F1 score results. We chose to examine these positions because they are the most used in the literature.

**Table 1:** Description of features extracted from the raw data.

Feature		Description
**Time**		
	Mean of vector magnitude	Sample mean of the VM^a^ in the window
	SD of vector magnitude	SD of VM in the window
	Mean angle of acceleration relative to vertical on the deice	Sample mean of the angle between x-axis and VM in the window
	SD of the angle of acceleration relative to vertical on the device	Sample SD of the angles in the window
	Covariance	Covariance of the VM in the window
	Skewness	Skewness of the VM in the window
	Kurtosis	Kurtosis of the VM in the window
	Entropy	Entropy of the VM in the window
	Coefficient of variation	SD of VM in the window divided by the mean, multiplied by 100
	Corr(x,y)	Correlation between x-axis and y-axis
	Corr(y,z)	Correlation between y-axis and z-axis
	Corr(x,z)	Correlation between x-axis and z-axis
**Frequency**		
	Percentage of the power of the VM that is in 0.6-2.5 Hz	Sum of moduli corresponding to frequency in this range divided by sum of moduli of all frequencies
	Dominant frequency of VM	Frequency corresponding to the largest modulus
	Fraction of power in VM at dominant frequency	Modulus of the dominant frequency or sum of moduli at each frequency

^a^VM: vector magnitude.

### Model Evaluation

We calculated the performance metrics of the models by comparing the model-based predicted values with the measured values. For the performance of the individual activity recognition model, we calculated the total accuracy of the model. For activity category recognition, we used the balanced accuracy metric to report model performance because of the class imbalance (ratio of the majority class to minority class being much smaller than 1) across activities. Balanced accuracy is defined as the mean of sensitivity and specificity metrics [47,48]. For MET estimation, we used the predicted and measured values to calculate the root mean square error (RMSE). The results were summarized into 3 major categories: the most accurate combination, the most accurate placement performance, and the most efficient combination. The latter was defined as the fewest number of sensors that provide a similar performance to the most accurate combination, with less than a 10% decrease in performance compared with the most accurate combination. For visualization purposes, the difference in the balanced accuracy of body placement/s compared with the accuracy derived from all 5 sensors was plotted. They were grouped by the number of body placements and ranked to simplify the visual comparisons. To compare across figures, the absolute value of the individual balanced accuracy was also added to the illustration.

## Results

The study enrolled 93 older adults (mean age 72.2, SD 7.1 years). The sample was balanced across gender, was mostly non-Hispanic White, and had comorbidities similar to those of the general population. Table 2 presents the descriptive characteristics of the participants. The participants completed 2013 tasks. The median number of tasks completed was 26 out of 32 tasks (Multimedia Appendix 1). Stair ascent had the lowest amount of complete data (n=43) and leisure walk had the most complete data (n=82). The reasons for missing information included not reaching a steady-state metabolic rate, invalid data from one or more monitors, unable to complete the task for at least 4 minutes, missed visits, or provided only partial data because the participant withdrew from the study.

**Table 2:** Participant characteristics (n=93).

Characteristics			Values
Age (years), mean (SD)			72.17 (7.02)
Female, n (%)			47 (51)
BMI (kg/m^2^), mean (SD)			28.18 (4.92)
**Race or ethnicity, n (%)**			
	Non-Hispanic White	83 (89)	
	Non-Hispanic Black	8 (9)	
	Non-Hispanic Asian	1 (1)	
	Hispanic	2 (2)	
Education (≥16 years), n (%)			15 (16)
Married or in a relationship, n (%)			52 (56)
Live alone, n (%)			30 (32)
Household income (≥US $15,000), n (%)			66 (71)
Self-rated health (≥good), n (%)			87 (94)
**Self-reported conditions, n (%)**			
	Former or current smoker	37 (40)	
	Hypertension	45 (48)	
	Hypercholesterolemia	39 (42)	
	Diabetes	19 (20)	
	Chronic pulmonary disease	10 (11)	
	Heart attack, myocardial infarction	8 (9)	
	Cancer	27 (29)	
	Depression	10 (11)	
	Stroke	4 (4)	
	Osteoarthritis	11 (12)	
Total moderate physical activity (min/week)^a^			93.50
**Walking speed (min per second), mean (SD)**			
	Leisure pace^b^	1.29 (0.26)	
	Rapid pace^c^	1.41 (0.25)	

^a^Data included for 77 participants.

^b^Data included for 91 participants.

^c^Data included for 85 participants.

**Figure 3:**
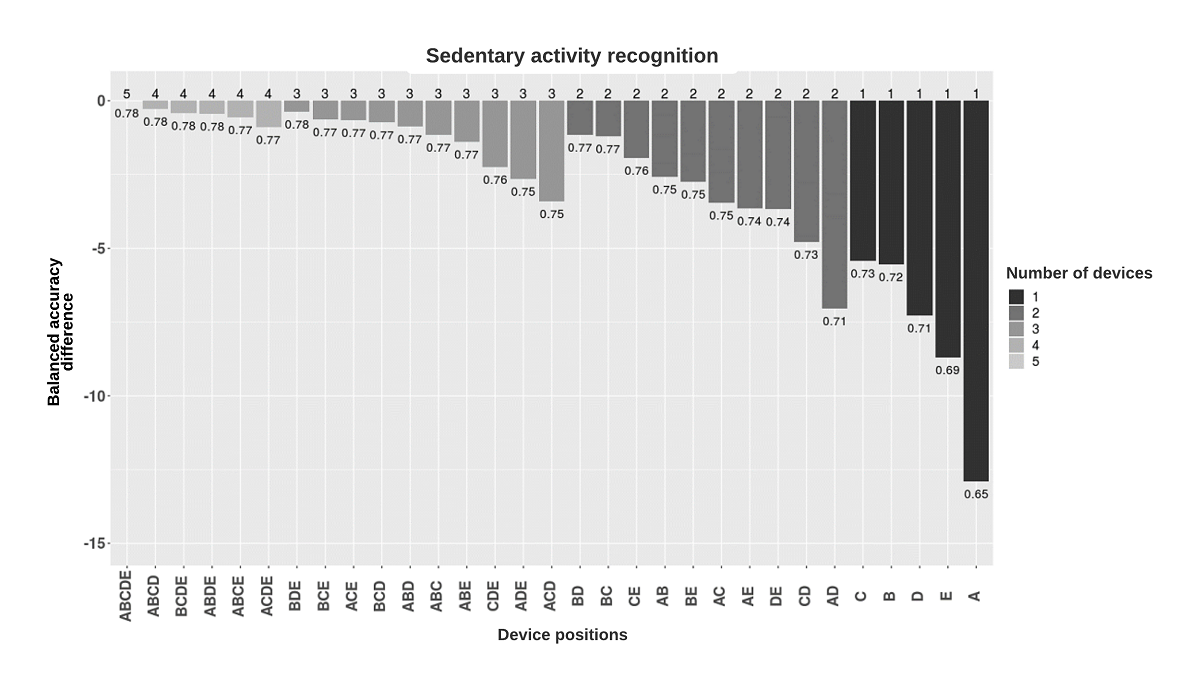
Balanced accuracy performance of sedentary activity classification models based on the device placement combinations. Models were grouped by the number of devices used and, in each group, were sorted by decreasing balanced accuracy (rounded). Y-axis shows the difference between the balanced accuracies of the different combinations and the five-placement combination. Numbers in the plot show the balanced accuracies of each placement combination. A: ankle; B: upper arm; C: hip; D: thigh; E: wrist.

**Figure 4:**
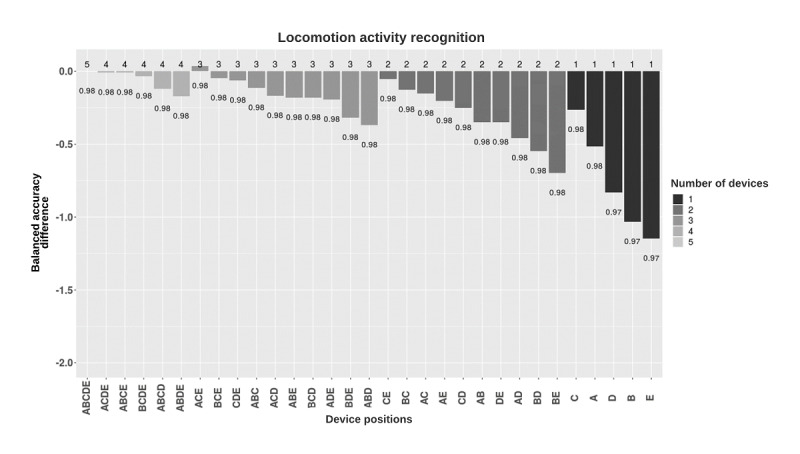
Balanced accuracy performance of locomotion activity classification models based on the device placement combinations. Models were grouped by the number of devices used and, in each group, were sorted in decreasing balanced accuracy (rounded). Y-axis shows the difference between the balanced accuracies of the different combinations and the five-placement combination. Numbers in the plot show the balanced accuracies of each placement combination. A: ankle; B: upper arm; C: hip; D: thigh; E: wrist.

**Figure 5:**
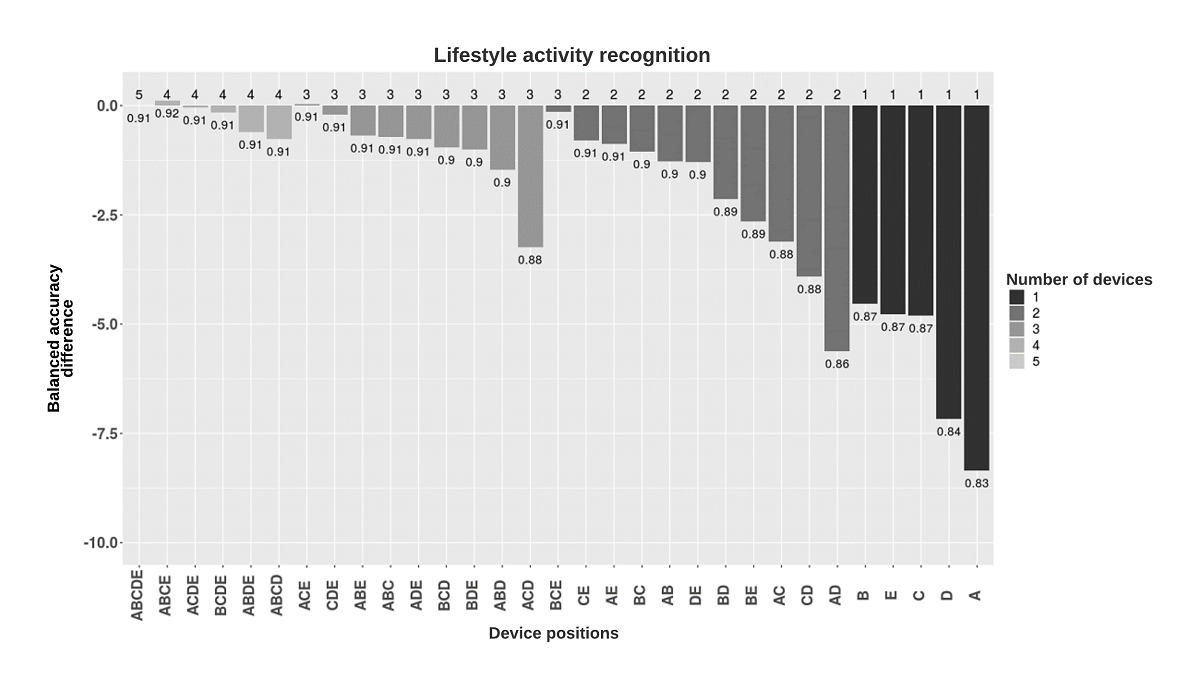
Balanced accuracy performance of lifestyle activity classification models based on the device placement combinations. Models were grouped by the number of devices used and, in each group, were sorted in decreasing balanced accuracy (rounded). Y-axis shows the difference between the balanced accuracies of the different combinations and the five-placement combination. Numbers in the plot show the balanced accuracies of each placement combination. A: ankle; B: upper arm; C: hip; D: thigh; E: wrist.

Models were also tested for categorizing sedentary, locomotion, and lifestyle activities (Figures 3-5). For sedentary behavior recognition, the combination of all accelerometers resulted in the best performance (balanced accuracy 0.78). Hip-worn placement provided the best performance among the single-placement models (balanced accuracy 0.73). The ankle-worn placement resulted in the worst performance (balanced accuracy 0.65). Multimedia Appendices 2 and 3 illustrate confusion matrices of the hip and wrist positions revealing that strength exercise and yoga, both partially done in a sitting position, were mislabeled as being sedentary activities, which caused significant overall misclassification.

For locomotion activity recognition, the combination of all placements resulted in the best performance (balanced accuracy 0.98). Hip-worn placement provided the best performance among the single-placement models (balanced accuracy 0.98). Classifiers trained separately on data from ankle-worn, wrist-worn, arm-worn, and thigh-worn placement also resulted in high performance (balanced accuracy 0.97-0.98; Figure 4).

For lifestyle activity recognition, the combination of data from ankle-worn, arm-worn, hip-worn, and wrist-worn placements resulted in the best performance (balanced accuracy 0.92). The combination of data from all placements resulted in high performance (balanced accuracy 0.91). Classifiers trained on data from arm-worn placements, similar to hip-worn and wrist-worn placements, provided the best performance among the single-placement models (balanced accuracy 0.87), whereas ankle-worn placement resulted in the lowest performance (balanced accuracy 0.83; Figure 5).

**Figure 6:**
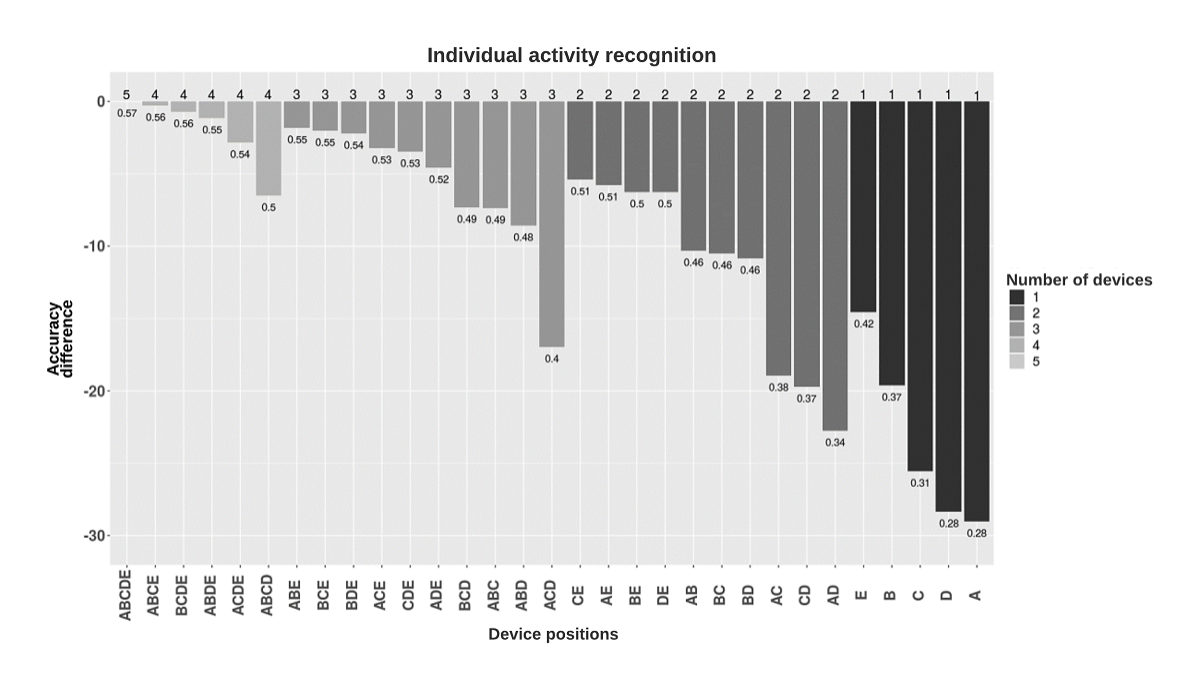
Accuracy performance of individual activity classification models based on the device placement combinations. Models were grouped by the number of devices used and, in each group, were sorted in decreasing accuracy (rounded). Y-axis shows the difference between the accuracies of the different combinations and the five-placement combination. Numbers in the plot show the accuracy of each placement combination. A: ankle; B: upper arm; C: hip; D: thigh; E: wrist.

The individual activity recognition models with all placements resulted in a relatively low accuracy of 0.57 (Figure 6). Wrist-worn placement provided the best performance among the single-placement models (accuracy 0.42). Classifiers trained separately on data from the ankle-worn placement, similar to thigh-worn placement, resulted in the worst performance (accuracy 0.28; Figure 6).

**Figure 7:**
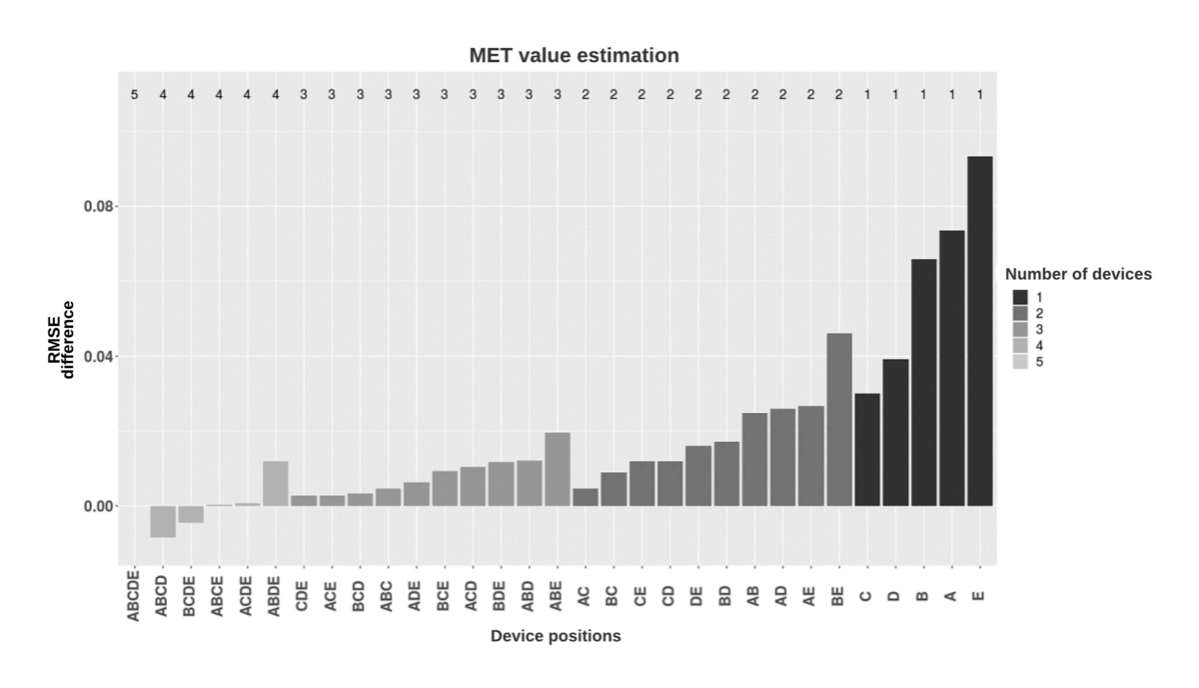
Root mean square error (RMSE) score performance of met value estimation models based on the device placement combinations. Models were grouped by the number of devices used and, in each group, were sorted in increasing RMSE (rounded). Y-axis shows the difference between the RMSE values of the different combinations and the five-placement combination. A: ankle; B: upper arm; C: hip; D: thigh; E: wrist; MET: metabolic equivalent; RMSE: root mean square error.

Energy expenditure accuracy was evaluated using the MET RMSE of the predicted versus measured values (Figure 7). In general, models trained using the combination of data from all 5 placements resulted in an RMSE of 0.88 METs. Hip-worn and thigh-worn placements provided the lowest RMSE of 0.91 METs among the single body placements. Overall, there was a slight reduction in RMSE when additional accelerometer placement was added to the model.

Table 3 summarizes the results according to the positions deemed most accurate, best single placement, and most efficient combination. In general, the most accurate combination contained data from all 5 body positions, but the most accurate placement was often very similar and sometimes better than combinations. The hip and wrist positions appeared to be the most efficient combinations, but models were able to recognize individual activities only with chance probability.

**Table 3:** Guideline table to determine the needed number and placement of the wearable accelerometer for each task.

Task	Most accurate combination	Most accurate single placement	Most efficient combination^a^
Sedentary activity detection (balanced accuracy)	All 5 placements (0.78)	Hip (0.73)	Hip (0.73)
Locomotion activity detection (balanced accuracy)	All 5 placements (0.98)	Hip (0.98); ankle (0.98)	Hip (0.98)
Lifestyle activity detection (balanced accuracy)	Ankle+upper arm+hip+wrist (0.92)	Upper arm (0.87); wrist (0.87); hip (0.87)	Wrist (0.87)
Individual activity recognition (accuracy)	All 5 placements (0.57)	Wrist (0.42)	Hip+wrist (0.51)
MET value estimation (root mean square error)	Ankle+upper arm+hip+thigh^b^ (0.87)	Hip (0.91); thigh (0.91)	Hip+wrist (0.89)

^a^The most efficient combination was defined as the fewest number of sensors that provide a similar performance to the most accurate combination while considering usability. Similar performance was defined as a difference ≤10% of the most accurate combination. We considered the most-to-least usable placements to be wrist>hip>ankle>arm>thigh. Thus, if the performance difference was less than 10%, then the most usable placement was chosen as the most efficient. Best and worst performance refer to best and worst performance according to their balanced accuracy (best: highest balanced accuracy; worst: lowest balanced accuracy).

^b^The performance of the combination with the best performance (0.87) was very close to that of the combination with all 5 placements (0.88).

## Discussion

### Principal Findings

We compared the performance of activity recognition models based on different combinations of 5 accelerometer placements on 32 activities of daily life. We considered single-sensor and multisensor placement on the wrist, hip, ankle, upper arm, and thigh. Our results show that the models achieved the best performance in the classification of locomotion activities and lifestyle activities (balanced accuracies 0.98 and 0.91 for the all five-sensor combination, respectively), followed by the classification of sedentary activity (balanced accuracy 0.78). The correct labeling of individual activities was low (accuracy 0.57). Interestingly, increasing the number of accelerometer placements had very limited improvement in the classification accuracy of locomotion and lifestyle activities as well as estimating MET values.

There are also noteworthy results from locomotion and sedentary tasks. The accuracy of locomotion activity recognition was similar across all the placements, and only minor differences were found between the combinations (approximately 1%). It is worth mentioning that the wrist-worn accelerometer had relatively lower performance, which is potentially due to the locomotor-like hand movements observed in other nonlocomotor tasks (eg, washing windows and yard work). Nonetheless, even a single body placement would likely suffice for locomotion activities. Detecting sedentary tasks had low accuracies, although the five-sensor combination provided a 7%-20% increase in balanced accuracy compared with several single placements. Additional analyses demonstrated that the misclassification rate was higher for sedentary activities than for nonsedentary activities. This may be caused by an imbalance in the data collected; sedentary tasks comprise only 4 out of 32 activities and result in only 6% of the total epochs. Another potential reason might be the similarity of some of the nonsedentary and sedentary activities. Confusion matrices of individual activity recognition models show that strength exercise and some stretching and some yoga, which were performed in a sitting position for a significant amount of time, contained most of the error (approximately 25%-76% for the hip and 40%-50% for the wrist). These activities are not traditionally considered to be sedentary behavior but are often performed in a sitting position (confusion matrices presented in Multimedia Appendices 2 and 3).

Historically, the hip position has been the most common and well-validated accelerometer placement. Some studies have investigated the performance of classifiers using data from other sensor placements, such as the ankle and wrist [22,25,49]. However, few studies have systematically examined the accuracy differences between individuals and combinations of different body placements [50,51]. The results published by Arif and Kattan [50] demonstrated in a cohort of 9 young adults that body placement differences between the wrist, chest, and ankle were relatively small in terms of overall accuracy when classifying 12 activities (best overall F-measure for wrist placement: 93.9%, for ankle placement: 92.2%, and for chest placement: 93.9% vs for combined placements: 98.2%). Similar findings have been reported by Gao et al [51], where the following 4 placement positions were compared: chest, underarm, waist, and thigh to identify 5 different activities performed by 8 older adults. They reported accuracies ranging from 81.9% to 92.8% for single-placement classifiers and 83.2%-96.4% for multisensor classifiers. These 2 studies were consistent with the finding that additional accelerometers improve performance in detecting the physical activity type. This study increases this initial knowledge with a much larger sample size of older adults who performed an ample number of activities with and without overlapping movement patterns. Although more generalizable, the large sample size likely introduced more variability in movement patterns, making it more challenging to find a single common classifier appropriate for all people. As such, the lower performance for activity recognition observed in this study might test the limits of the predictive capacity for machine learning models, such as random forest, when applied across a diverse population.

A MET RMSE of 0.88 was achieved across all activities. Previous studies using data from accelerometer devices worn on the hip and wrist have shown similar results for the prediction of METs, with RMSE values of 1.00-1.22 [45,52,53]. For a single placement, the hip and thigh positions provided the lowest RMSE values. Increasing the number of placements only slightly enhanced the RMSE (from 3% to 9%). Our results also show that adding 2 or more accelerometers provides a small enhancement in prediction. Previous studies with a smaller number of activities had similar performance in MET estimation—1.0 METs and 1.2 METs using data collected from wrist and hip placements [42,45]. Our slightly better performance might be because of a large range of activities that enhanced MET distribution.

We believe that our work constitutes one of the largest accelerometer-based validation studies in older adults. Data were collected at a high resolution, and there were a large number of activities included and 5 body placements. This resulted in a large number of pairwise (location and sensor) combinations. A limitation of this study is that data were collected in controlled laboratory settings, which is an appropriate initial step in a validation framework [54]. The next step is to collect data in free-living settings with more fluid transitions between tasks, which is more reflective of actual movement. Another limitation of the study was that not all activities were performed by all participants (Multimedia Appendix 1). However, the final number of participants with complete data for each activity was sufficient to assess the accuracy of individual body positions and their combinations. Another limitation of the study was that the performance ranking and conclusions were based on random forest models and might change when using other machine learning models. We used the random forest model because it was found to be the best performing in our previous study [41]. A subsequent analysis is required to validate whether the choice of machine learning model will affect the classification performance. Finally, our population included community-dwelling older volunteers to generalize to this population. Although this sample had common comorbidities such as diabetes, hypertension, and cancer history, we did not actively recruit people who had specific ambulatory deficits that would likely impact the results. Existing work in these specialized populations shows that knowledge from nonambulatory, impaired (eg, healthier) adults transfers with poor accuracy [55]. Thus, this study is limited to community-dwelling older adults without overt ambulatory deficits.

### Conclusions

The results from this work suggest that additional accelerometer devices only slightly enhance activity recognition accuracy and MET estimation in older adults. However, no single or combination of accelerometer placement appeared to be significantly better than the others. Therefore, using a single accelerometer placement appears to provide sufficient performance for labeling general activity categories and estimating energy expenditure. Researchers and practitioners should consider performance accuracy in the context of participant burden and the potential extra benefits gained in particular positions.

## Appendix

Multimedia Appendix 1 Activity characteristics (four sedentary activities, six locomotion activities, and 22 lifestyle activities). RPE: rating of perceived exertion.

Multimedia Appendix 2 Confusion matrix for individual activity recognition using data from hip placement.

Multimedia Appendix 3 Confusion matrix for individual activity recognition using data from wrist placement.

## Abbreviations

CO_2_: carbon dioxide
MET: metabolic equivalent
O_2_: oxygen
RMSE: root mean square error
VO_2_: oxygen consumption
