# Supplementary material for: The Effect of Sensor Placement and Number on Physical Activity Recognition and Energy Expenditure Estimation in Older Adults: Validation Study
Source: JMIR Mhealth Uhealth. 2021 May 3;9(5):e23681. doi: 10.2196/23681 (PMC8129874; doi:10.2196/23681)
Supplement: Multimedia Appendix 1 [file mhealth_v9i5e23681_app1.docx]

Supplimentary Table 1: Activity characteristics (four sedentary activities, six locomotion activities, and 22 lifestyle activities). RPE: rating of perceived exertion.

| Activity | Sedentary | Locomotion | Lifestyle | Length (minutes) | Number of participants |
| --- | --- | --- | --- | --- | --- |
| Vacuuming | no | no | yes | 419.2 | 63 |
| Computer work | yes | no | no | 117.3 | 58 |
| Home maintenance | no | no | yes | 391.2 | 62 |
| Washing windows | no | no | yes | 384.0 | 62 |
| Heavy lifting | no | no | yes | 317.9 | 53 |
| Replacing bed sheets | no | no | yes | 392.5 | 62 |
| Ironing | no | no | yes | 388.8 | 62 |
| Stretching/Yoga | no | no | yes | 324.8 | 61 |
| Trash removal | no | no | yes | 394.1 | 63 |
| Mopping | no | no | yes | 380.0 | 61 |
| Sweeping | no | no | yes | 408.3 | 65 |
| Stair descent | no | yes | no | 114.9 | 57 |
| Stair ascent | no | yes | no | 127.7 | 43 |
| Yard work | no | no | yes | 398.1 | 75 |
| Standing still | yes | no | no | 174.9 | 54 |
| Preparing and serving a meal | no | no | yes | 387.2 | 65 |
| Washing dishes | no | no | yes | 373.3 | 65 |
| Straightening up/dusting | no | no | yes | 393.6 | 65 |
| Dressing | no | no | yes | 356.8 | 58 |
| TV watching | yes | no | no | 89.6 | 52 |
| Strength exercise | no | no | yes | 358.4 | 57 |
| Lying | yes | no | no | 239.7 | 59 |
| Light gardening | no | no | yes | 444.8 | 78 |
| Shopping | no | no | yes | 387.2 | 62 |
| Personal care | no | no | yes | 373.9 | 65 |
| Digging | no | no | yes | 354.4 | 71 |
| Unloading and storing dishes | no | no | yes | 383.7 | 65 |
| Laundry | no | no | yes | 381.1 | 62 |
| Leisure walk | no | yes | no | 490.7 | 82 |
| Rapid walk | no | yes | no | 452.3 | 80 |
| Walking @ 1RPE | no | yes | no | 236.5 | 65 |
| Walking @ 5RPE | no | yes | no | 209.9 | 61 |
